# Genomic Basis of Lifestyle Divergence in Rice-Associated *Burkholderia*: From Pathogenesis to Plant Growth Promotion

**DOI:** 10.3390/ijms27114730

**Published:** 2026-05-24

**Authors:** Andrews Danso Ofori, Zohreh Nasimi, Frank Kwekucher Ackah, Muhammad Irfan Ahmed, Yaoting Yan, Wang Li, Abdul Ghani Kandro, Kazunori Okada, Keiichi Mochida, Yoshiteru Noutoshi, Aiping Zheng

**Affiliations:** 1 State Key Laboratory of Crop Gene Exploration and Utilization in Southwest China, Sichuan Agricultural University, Chengdu 611130, Chinaz.nasimi@sicau.edu.cn (Z.N.);; 2 Department of Plant Pathology, Rice Research Institute, Sichuan Agricultural University, Chengdu 611130, China; 3 Department of Crop Science, School of Agriculture and Natural Sciences, University of Cape Coast, Cape Coast 00233, Ghana; frank.ackah@ucc.edu.gh; 4 Agro-Biotechnology Research Center, The University of Tokyo, Bunkyo-ku 113-8657, Japan; kazunoriokada@gmail.com; 5 Bioproductivity Informatics Research Team, RIKEN Center for Sustainable Resource Science, Yokohama 230-0045, Japan; keiichi.mochida@riken.jp; 6 Microalgae Production Control Technology Laboratory, RIKEN Baton Zone Program, RIKEN Cluster for Science, Technology and Innovation Hub, Saitama 351-0198, Japan; 7 Kihara Institute for Biological Research, Yokohama City University, Yokohama 244-0813, Japan; 8 School of Information and Data Sciences, Nagasaki University, Nagasaki 852-8521, Japan; 9 Graduate School of Environmental and Life Science, Okayama University, Okayama 700-8530, Japan

**Keywords:** *Burkholderia*, comparative genomics, virulence factors, secretion systems, CAZymes, plant-microbe interactions, biocontrol, pathogenicity, protein–protein interaction networks, rice sheath blight

## Abstract

The genus *Burkholderia* encompasses both plant pathogenic and beneficial species, yet the genomic determinants underlying this lifestyle divergence remain poorly understood. Using 16S rRNA sequencing of 100 rice cultivars, our companion study demonstrated that resistant varieties are enriched in beneficial *Burkholderiaceae*, leading to the isolation of three phenotypically contrasting strains. Here, we present comparative genomic analyses of non-pathogenic biocontrol strain *Burkholderia vietnamiensis* J14EpLeaf2 and pathogenic strains *Burkholderia gladioli* A1EpSeed5 and *Burkholderia cepacia* J14Eple. Pathogenic strains possess significantly larger genomes (8.36–8.46 Mb) enriched in mobile genetic elements compared to the streamlined 6.95 Mb genome of *B. vietnamiensis*. CAZyme analysis revealed broader repertoires of glycoside hydrolases and polysaccharide lyases in pathogens, consistent with enhanced plant cell wall degradation. *B. gladioli* possesses a complete T3SS and expanded T6SS with 301 predicted effectors, while *B. cepacia* lacks structural T3SS genes but harbors 271 candidate effectors predicted to be secreted via alternative secretion pathways, compared to 180 in *B. vietnamiensis*. Notably, *B. cepacia* harbors cystic fibrosis-associated markers (cable pili, ZmpA/ZmpB), raising significant biosafety concerns that preclude its agricultural application. LC-MS validated IAA, ornibactin, and AHL production in *B. vietnamiensis*, supporting its plant growth-promoting and biocontrol functions. Computational PPI networks predicted distinct interaction landscapes requiring experimental validation. This study provides a genomic framework for distinguishing pathogenic from beneficial *Burkholderia* and supports *B. vietnamiensis* as a safe biocontrol agent while cautioning against *B. cepacia* J14Eple.

## 1. Introduction

Rice (*Oryza sativa* L.) is a fundamental crop that feeds more than half the world’s population, accounting for over 23% of global caloric intake [1,2]. Nonetheless, rice production is drastically threatened by various biotic stresses, with sheath blight caused by *Rhizoctonia solani* AG1-IA among the utmost destructive pathogens, causing yield losses of 10–30% and up to 50% under favorable conditions [3,4]. While the phyllosphere microbiome plays vital roles in plant health, the genus *Burkholderia* has transpired as particularly significant due to its ecological duality and metabolic versatility [5].

The genus *Burkholderia* embodies a wide range of metabolic capabilities and ecological adaptability, housing both harmful pathogens and beneficial plant-associated bacteria [6]. Some species are classified as opportunistic plant and human pathogens, including members of the *Burkholderia cepacia* complex (Bcc), *B. gladioli*, and *B. glumae* [6,7]. These pathogenic strains cause destructive crop diseases and severe lung infections in patients with cystic fibrosis [8,9]. In contrast, other *Burkholderia* species, particularly diazotrophic and endophytic strains, are known to produce antibiotics, compete for nutrients and niches, and induce systemic resistance to promote plant growth and shield hosts from fungal diseases [10,11]. *Burkholderia vietnamiensis*, for example, has been documented as a plant growth-promoting bacterium capable of nitrogen fixation and biocontrol [12].

The dual reputation of *Burkholderia* renders it a particularly intriguing target for biocontrol development, as its established ability to exert potent antagonism must be weighed against a meticulous evaluation of biosafety and host range [13]. Strain-level assessment is critical, as even within a single named species, some strains are adapted to human or plant infection while others are primarily plant-associated and comparatively benign [14,15].

Using 16S rRNA amplicon sequencing of 100 rice cultivars with varying susceptibility to *R. solani*, our companion study [16] demonstrated that resistant varieties harbor significantly more diverse bacterial communities (3230 OTUs; 2064 unique) than susceptible cultivars (599 OTUs; 36 unique). Resistant varieties were specifically enriched in beneficial *Burkholderiaceae*, *Bacillaceae*, and *Pseudomonadaceae*, while *Sphingobacteriaceae* and *Enterobacteriaceae* predominated in susceptible varieties. These findings provided the ecological rationale for isolating and phenotypically characterizing *Burkholderia* strains from resistant rice varieties, leading to the identification of *B. vietnamiensis* J14EpLeaf2 as a potent biocontrol agent with 77% growth inhibition of *R. solani*, broad-spectrum antifungal activity, and multiple plant growth-promoting traits [16]. Phenotypic biosafety assessment confirmed that *B. vietnamiensis* is non-pathogenic to rice and tobacco, while *B. gladioli* A1EpSeed5 and *B. cepacia* J14Eple caused rapid necrosis and chlorosis.

Previous work has confirmed that the rice phyllosphere harbors functionally heterogeneous *Burkholderia* strains, underscoring the significance of understanding the genetic foundations underlying their divergent phenotypes. A comparative genomic analysis of rice-associated *Burkholderia* offers a robust framework for elucidating the molecular factors that regulate pathogenic and beneficial lifestyles [17,18].

In this study, we tested the hypothesis that pathogenic and beneficial *Burkholderia* strains from the same ecological niche can be distinguished by specific genomic features. Specifically, we predicted that: (i) pathogenic strains would possess larger genomes enriched in mobile genetic elements, genomic islands, and prophages; (ii) pathogenic strains would harbor complete Type III secretion systems (T3SS) and expanded repertoires of virulence factors and CAZymes; (iii) the beneficial *B. vietnamiensis* would be enriched for plant-growth-promotion genes, secondary metabolite biosynthetic clusters, and quorum quenching enzymes; and (iv) *B. cepacia* J14Eple would carry markers of opportunistic pathogenicity relevant to human health.

This study is accompanied by a companion paper [16] reporting the 16S rRNA microbiome analysis of 100 rice cultivars, isolation of *Burkholderia* strains, and comprehensive phenotypic characterization of biocontrol and plant growth-promoting activities. Here, we present the comparative genomic analysis of three representative strains to elucidate the molecular basis of their divergent lifestyles.

## 2. Results

### 2.1. Genome Size and General Features

Genome assembly metrics showed notable variation in genome size and assembly continuity across the three *Burkholderia strains* (Table 1). The genome sizes of all strains ranged from 6.95 Mb to 8.46 Mb, with the largest *B. cepacia* J14Eple genome (8.46 Mb) and the smallest *B. vietnamiensis* J14EpLeaf2 genome (6.95 Mb). The assembly continuity of the three genomes also differed. *B. cepacia* had the highest contiguity with the lowest number of scaffolds (56) and the highest N50 value (335,300 bp), while *B. gladioli* had the most fragmented assembly. *B. vietnamiensis* had an intermediate-contiguity assembly profile.

The GC content of all the genomes was high (66.84–67.9%), which is consistent with the high GC content characteristic of the genus. The larger genomes and greater degree of complexity, as evidenced by differences between *B. cepacia* and *B. gladioli*.

### 2.2. Mobile Genetic Elements and Genomic Plasticity

The results of the comparative analysis of mobile genetic elements among the *Burkholderia* species suggest that *B. cepacia* has the most plastic genome of the three species analyzed (see Table 2). With 19 genomic islands, *B. cepacia* shows evidence of greater levels of horizontal gene transfer and genomic diversification than the other two Burkholderia species analyzed—with *B. gladioli* and *B. vietnamiensis* each containing 14 and 12 genomic islands, respectively.

The number of prophages present in the *Burkholderia* species also differed between the three strains, with *B. gladioli* containing the greatest number of prophages (5), whereas *B. cepacia* and *B. vietnamiensis* each contained only 2 prophage regions. 

The number of CRISPR loci also differed between the *Burkholderia* spp. *B. gladioli* has the greatest number of CRISPR loci (12), followed by *B. vietnamiensis* (5), and *B. cepacia* (4).

### 2.3. Carbohydrate-Active Enzyme (CAZymes)

The comparative analysis of CAZyme repertoires revealed distinct patterns of distribution among the three *Burkholderia* strains, suggesting differences in predicted carbohydrate metabolism and plant cell wall modification capacity. Total CAZyme counts were highest for the GT class (i.e., glycosyltransferases) across all of the strains; *B. vietnamiensis* J14EpLeaf2 had the highest total GT count (114 genes) followed closely by *B. cepacia* J14Eple (106 genes) and then *B. gladioli* A1EpSeed5 (102 genes). GHs (i.e., glycoside hydrolases), which are primarily associated with polysaccharide degradation, were also represented significantly in each strain; *B. gladioli* had the highest number (81 genes) followed closely by *B. cepacia* (79 genes) and then *B. vietnamiensis* (58 genes). CBMs (i.e., carbohydrate-binding modules) exhibited relatively similar distribution patterns across the three strains, with *B. gladioli* (35 genes) and *B. cepacia* (34 genes) having modestly more than *B. vietnamiensis* (28 genes). CEs (i.e., carbohydrate esterases) were moderate in the strains, with *B. cepacia* exhibiting the highest number (16 genes), while *B. vietnamiensis* had 11 genes and *B. gladioli* had 10 genes.

**Figure 1:**
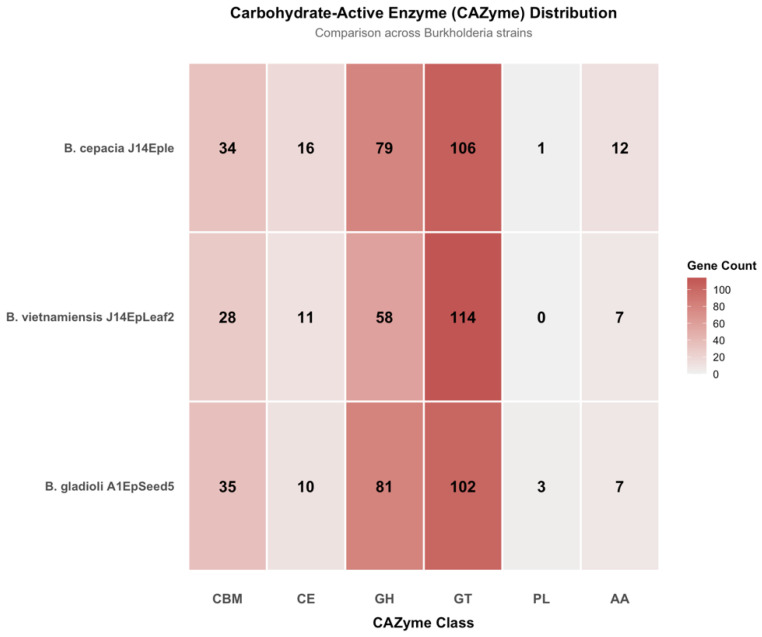
Comparative heatmap of carbohydrate-active enzyme (CAZyme) classes in rice-associated *Burkholderia* strains. GH: Glycoside Hydrolases, GT: Glycosyl Transferases, PL: Polysaccharide Lyases, CE: Carbohydrate Esterases, AA: Auxiliary Activities, CBM: Carbohydrate-Binding Modules. Color intensity reflects gene copy number within each class, ranging from light yellow (low abundance) to dark blue (high abundance).

Notably absent in *B. vietnamiensis*, PLs (i.e., polysaccharide lyases that macerate plant cell walls and degrade pectin) were present in low abundance in *B. gladioli* (3 genes) and in *B. cepacia* (1 gene). All three strains contained AA (i.e., auxiliary activity) enzymes that metabolize carbohydrates in an oxidative manner, with *B. cepacia* containing more AA enzymes (12 genes) than the other two strains (*B. vietnamiensis* 7 genes; *B. gladioli* 7 genes) (Figure 1). Detailed CAZyme family distributions and gene counts are provided in Supplementary Table S2.

### 2.4. Secretion Systems and T3SS Effectors

All three strains harbor secretion systems, albeit with differences in architecture and/or the number of genes (Table 3). *B. gladioli* possess a full T3SS (5 genes) and has the most extensive T6SS (14 genes) among the three strains. While *B. vietnamiensis* also has 7 T3SS-related genes, further pathway-level testing is necessary to determine whether these genes constitute a complete functional secretion system or represent a vestigial or repurposed system, as in other plant-associated bacteria. On the other hand, *B. cepacia* has no identifiable T3SS and relies on the T2SS (5 genes) and T6SS (6 genes) for protein secretion. Nonetheless, to predict effectors encoded by *B. cepacia*, 271 effector proteins were identified by EffectiveT3, relatively close to *B. gladioli* (301) but much higher than *B. vietnamiensis* (180) in terms of predicted genes. 

### 2.5. Secretory Protein Enrichment

**Figure 2:**
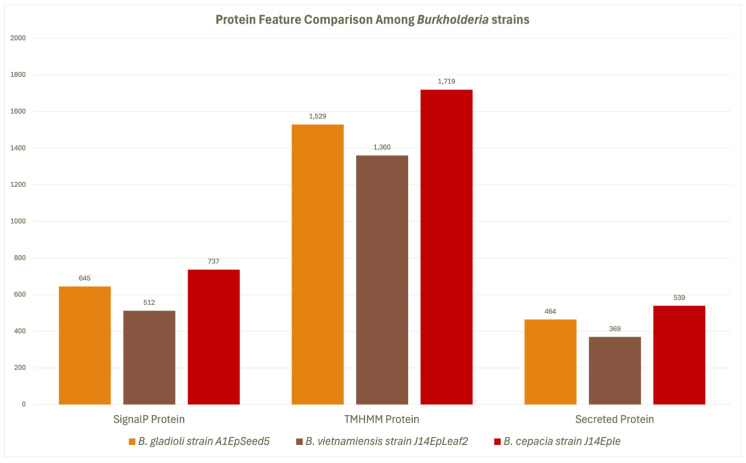
Protein feature distribution among *Burkholderia* strains.

The total number of proteins containing signal peptides, transmembrane domain(s) and those predicted to be secreted varies greatly between bacteria type (Figure 2). The bacterium *B. vietnamiensis* has the fewest proteins with signal peptides (512), transmembrane domains (1360), and predicted secretion signals (369). In contrast, pathogenic strains have higher total numbers of these proteins (*B. gladioli*; signal peptide: 645; trans-membrane: 1529; predicted secreted: 464; *B. cepacia*; signal peptide: 737; trans-membrane: 1719; predicted secreted: 539). GO and KEGG analyses were performed using secretory protein data (Section 2.12). Enrichment for *B. vietnamiensis* was found to be unique in the nitrogen compound transport and quorum sensing pathways, while *B. gladioli* and *B. cepacia* were enriched in iron acquisition functions and functions related to heme binding and efflux, respectively.

### 2.6. Quorum Sensing and Quorum Quenching Enzyme

All three strains of the bacteria contain genes for producing AHLs (acyl-homoserine lactones) and for regulatory proteins (LuxRs) that control their production. Only the strain *B. vietnamiensis* J14EpLeaf2 has the gene for penicillin V acylase, which may act as a quorum quenching enzyme (Table 4). The identification of AHLs such as C4-HSL, C10-HSL, and C12-HSL using LC-MS confirms that *B. vietnamiensis* produces AHLs in accordance with the presence of AHL synthase genes.

### 2.7. Virulence Factor Profile (VFDB)

**Figure 3:**
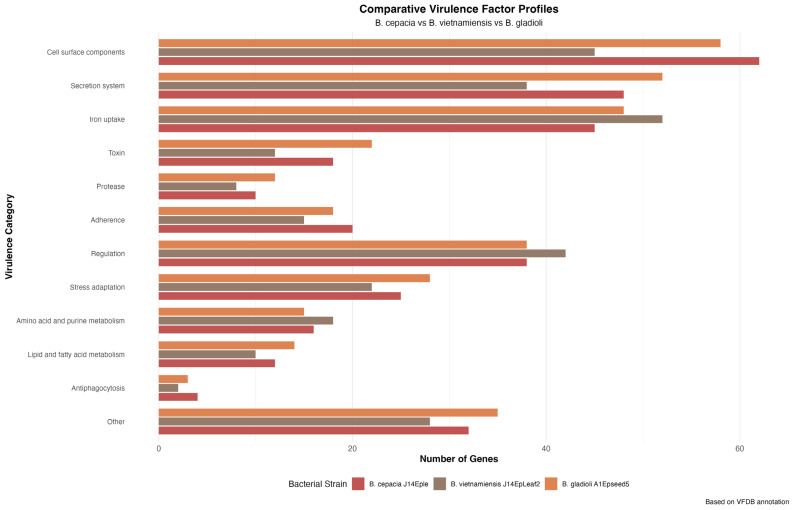
Comparative virulence factor profiles of rice-associated *Burkholderia* strains based on VFDB annotation. Stacked bar plot showing the distribution of virulence-associated genes across functional categories in *B. cepacia* J14Eple, *B. vietnamiensis* J14EpLeaf2, and *B. gladioli* A1Epseed5. *B. vietnamiensis* is enriched in motility, chemotaxis, biofilm formation, and iron uptake systems, reflecting adaptations for plant colonization. *B. gladioli* harbors classical virulence determinants, including LOS biosynthesis, capsule formation, urease, and phytotoxins. *B. cepacia* possesses the most extensive virulence arsenal, including cable pili, zinc metalloproteases ZmpA/ZmpB, complete Capsule I cluster, and multiple T6SS systems, consistent with its high-risk pathogenicity. Both pathogenic strains exhibit elevated counts in cell surface components, secretion systems, and toxins compared to the non-pathogenic *B. vietnamiensis*.

According to an analysis conducted using VFDB, both *B. gladioli* and *B. cepacia* have larger virulence gene repertoires than *B. vietnamiensis* (Table 5, Figure 3). The main finding was that there is a complete Capsule I cluster (over 20 genes) present in *B. gladioli*, a partial cluster in *B. cepacia*, and no Capsule I cluster in *B. vietnamiensis*. Lipooligosaccharide (LOS) biosynthesis pathways were complete in both pathogenic strains. A complete T3SS apparatus was identified only in *B. gladioli*; *B. cepacia* lacked structural T3SS genes, as confirmed by TnSS annotation. Phytotoxin-associated genes, including *cysC1*, coronatine, and syringomycin-related genes, were extensively present in *B. gladioli* but were absent or minimally represented in *B. vietnamiensis*. Additionally, urease subunits were present exclusively in the pathogenic strains. Complete VFDB virulence factor annotations by category are provided in Supplementary Table S3.

### 2.8. Opportunistic Pathogenicity Markers and Phenotypic Validation

The analysis of virulence factors related to cystic fibrosis demonstrated that *B. cepacia* J14Eple harbored the common virulence determinants of *Burkholderia* (Table 6), including genes for the cable pili and the secreted zinc metalloproteases ZmpA and ZmpB, characteristics of highly transmissible and high-risk Bcc lineages commonly found in cystic fibrosis patients. *B. vietnamiensis* and *B. gladioli* did not possess these characteristics. All three strains lacked sodC or VgrG-5, which are indicative of invasive disease, while all three strains contained the CF-associated O-antigen loci. Consistent with our previous studies [16], HR assays performed on *Nicotiana benthamiana* and pathogenicity testing of rice demonstrated that *B. gladioli* A1EpSeed5 and *B. cepacia* J14Eple produced necrotic lesions on tobacco plants within 24 h of inoculation and produced chlorosis and water-soaked lesions on rice blades, panicles, and sheaths within three days after inoculation. In contrast, *B. vietnamiensis* J14EpLeaf2 did not cause visible disease symptoms on either host plant.

### 2.9. Antimicrobial Resistance Profile

**Figure 4:**
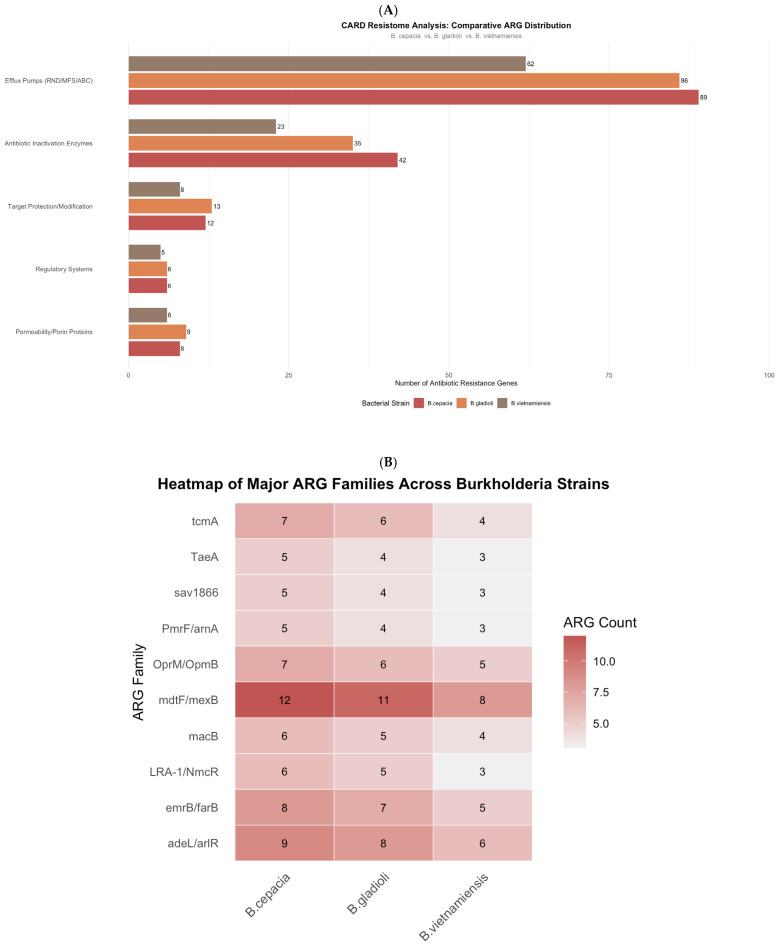
Comparative resistome analysis of *Burkholderia* strains. (**A**) Distribution of antibiotic resistance genes (ARGs) by functional category across *B. cepacia*, *B. gladioli* and *B. vietnamiensis*. Efflux pumps constitute the most abundant resistance mechanism in all three strains, with pathogenic *B. cepacia* and *B. gladioli* harboring substantially more genes in this category (89 and 86, respectively) compared with non-pathogenic *B. vietnamiensis* (62). Enrichment is also observed for antibiotic inactivation enzymes (42 and 35 versus 23). (**B**) Heatmap showing the abundance of the ten most prevalent ARG families across the three strains. The RND efflux pump families *mdtF*/*mexB* and *oprM*/*opmB*, together with the beta-lactamase families LRA-1/NmcR and the MFS efflux pump *emrB*/*farB*, are markedly expanded in the pathogenic isolates, whereas *B. vietnamiensis* consistently displays lower copy numbers across all families. Colour intensity reflects ARG copy number per strain.

A total of 387 putative antibiotic resistance genes (ARGs) were identified across the three genomes (Figure 4A). *B. cepacia* harbored the largest resistome (149 ARGs), followed by *B. gladioli* (140 ARGs) and *B. vietnamiensis* (98 ARGs). Efflux pump systems (primarily RND family) constituted the dominant resistance mechanism (60–62% of total ARGs). Beta-lactamase genes (LRA-1, NmcR, OXA variants) (Figure 4B) were enriched in pathogenic strains. Importantly, ARGs were predominantly intrinsic and chromosomally encoded, not associated with mobile genetic elements—a reassuring finding for biocontrol strain safety. Detailed antibiotic resistance gene categories and mechanisms are provided in Supplementary Table S4.

### 2.10. PHI-Base Phenotypye Distribution

**Figure 5:**
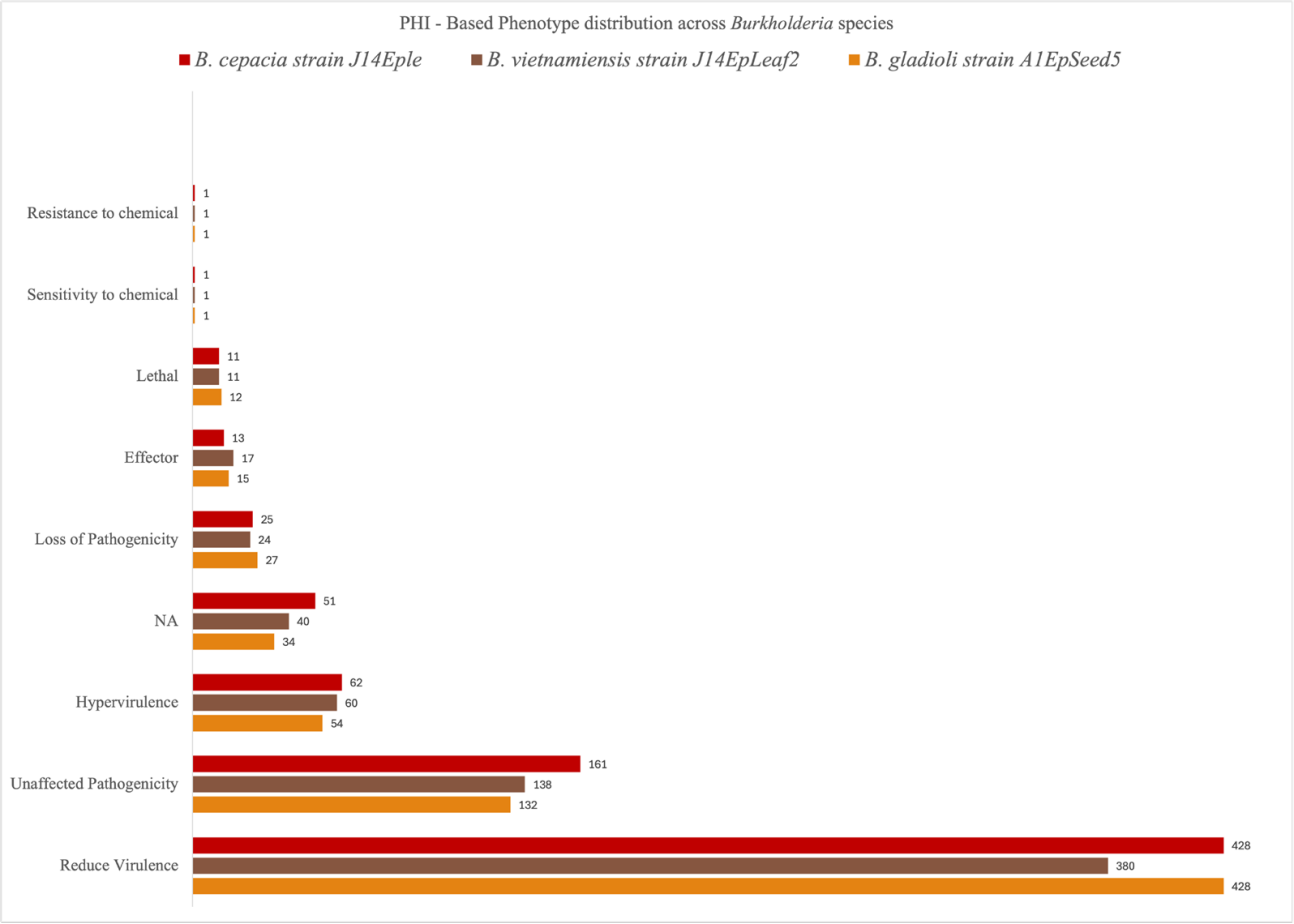
PHI-base phenotype distribution across *Burkholderia* species. Genes from *B. cepacia*, *B. vietnamiensis*, and *B. gladioli* were classified according to PHI-base phenotype categories. Bar plots show the number of genes associated with each phenotype, including reduced virulence, unaffected pathogenicity, hypervirulence, loss of pathogenicity, effector (plant avirulence determinant), lethal, and chemical sensitivity/resistance. Across all species, reduced virulence represents the dominant category, followed by unaffected pathogenicity. Minor categories, including effector and lethal phenotypes, are conserved at lower frequencies. Overall, *B. cepacia* and *B. gladioli* exhibit slightly expanded repertoires of virulence-associated phenotypes compared to *B. vietnamiensis*.

PHI-base annotation revealed that “reduced virulence” was the dominant phenotype category across all strains (380–428 genes) (Figure 5). Hypervirulence-associated genes were moderately represented, with slightly higher counts in *B. cepacia* (62) and *B. vietnamiensis* (60) compared to *B. gladioli* (54). Effector-related genes (plant avirulence determinants) were present in all three genomes (13–17 genes). Full PHI-base results are provided in Supplementary Table S5.

### 2.11. Secondary Metabolite Biosynthesis and LC-MS Validation

**Figure 6:**
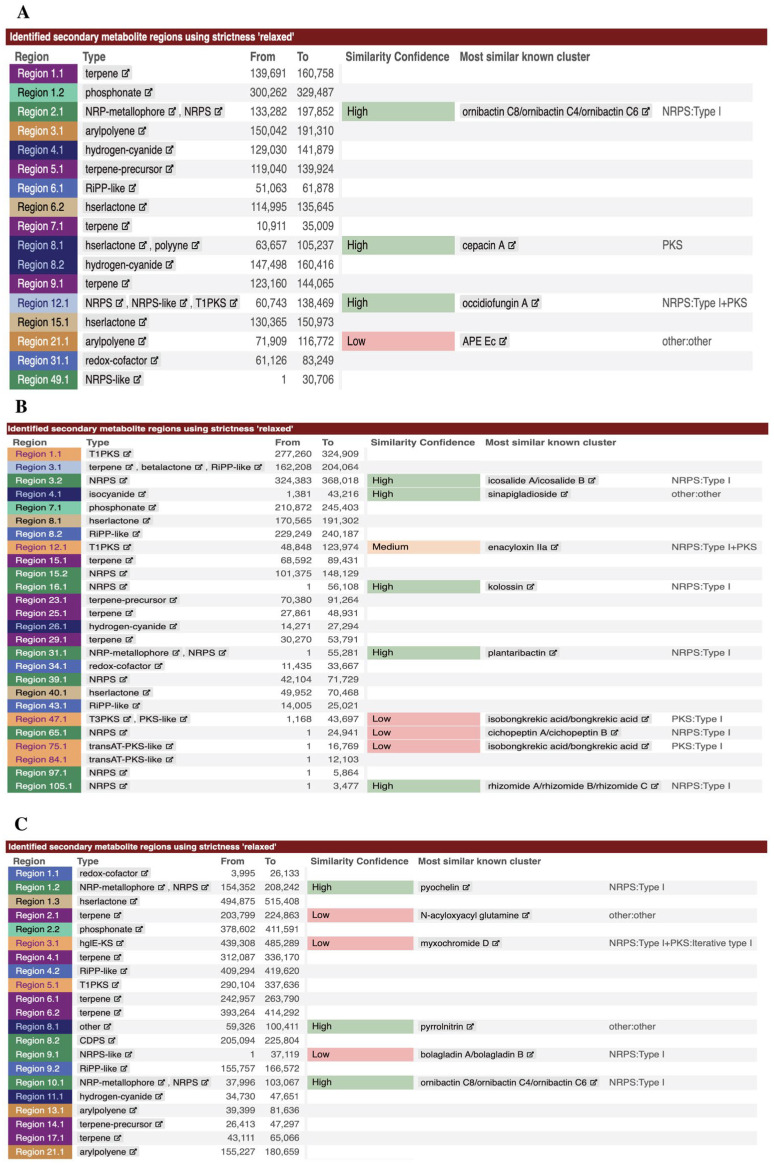
antiSMASH-predicted biosynthetic gene clusters. (**A**) *Burkholderia vietnamiensis* J14EpLeaf2: Predicted BGCs include terpene, hydrocarbon, aliphatic, oxygen, terpene precursor, RiPP-like, and sesquiterpenoid clusters. (**B**) *Burkholderia gladioli* A1EpSeed5: Predicted BGCs include T1PKS, terpene, hydrocarbon, isocyclic, RiPP-like, transAT-PKS-like, NRPS, and NRPS-methyltransferase clusters. (**C**) *Burkholderia cepacia* J14Eple: Predicted BGCs include redox-cofactor, NRPS-metallophore, hydrocarbon, terpene, RiPP-like, T1PKS, CDPS, NRPS-like, and terpene precursor clusters. High-confidence known clusters identified include pyochelin (Region 1.2 in (**C**)), pyrrolnitrin (Region 2.2 in (**C**)), and ornibactin (Region 2.6 in (**C**)). BGCs were predicted using antiSMASH (version 8.0.2) with relaxed detection strictness. Each colored block represents a distinct BGC, with colors indicating BGC type according to the antiSMASH legend.

antiSMASH analysis revealed multiple biosynthetic gene clusters (BGCs) in both species (Figure 6). *B. vietnamiensis* contained BGCs for ornibactin (siderophore), cepacin A (antifungal polyyne), and occidiofungin A (hybrid NRPS/PKS antifungal). *B. gladioli* contained a larger number of BGCs, including enacyloxin IIa, icosalide, kolossin, and plantaribactin.

LC-MS metabolite profiling was conducted for *B. vietnamiensis* J14EpLeaf2 and *B. gladioli* A1EpSeed5, as these strains were the primary focus of biocontrol and plant growth promotion assessment in the companion study [16]. *B. cepacia* J14Eple was excluded from LC-MS analysis given its early designation as a biosafety risk, which precluded its consideration as a biocontrol candidate and making detailed metabolite validation a secondary priority.

LC-MS metabolomics experimentally validated these predictions (Table 7 and Table 8). Indole-3-acetic acid (IAA) was detected in *B. vietnamiensis* (*m*/*z* 174.0564, RT 5.24 min, Level 1 identification), confirming auxin biosynthesis capability. This finding is consistent with phenotypic IAA production measured by the Salkowski assay, where *B. vietnamiensis* produced significantly higher IAA levels than *B. gladioli* in the presence of L-tryptophan (*p* < 0.001); [16]. The identification of ornibactin-derived metabolites in *B. vietnamiensis* supports the results of our previous work, which demonstrated the production of siderophores via the Chrome Azurol S (CAS) assay [16]. Various AHLs were also detected, including C4-HSL (*m*/*z* = 172.0968, RT = 1.52 min), C10-HSL (*m*/*z* = 272.1869, RT = 6.09 min) and C12-HSL (*m*/*z* = 314.2343, RT = 6.81 min), establishing that *B. vietnamiensis* has the ability to carry out quorum sensing. Antifungal substances were also identified; M83 (occidiofungin-like; *m*/*z* = 466.1702, RT = 4.56 min) was discovered in *B. vietnamiensis*, and virginiamycin M1 (enacyloxin-like; *m*/*z* = 544.2662) was found in *B. gladioli*.

Genome mining of *Burkholderia cepacia* J14EPLE using antiSMASH identified multiple biosynthetic gene clusters (BGCs) spanning diverse classes of secondary metabolites. These included non-ribosomal peptide synthetases (NRPS), polyketide synthases (PKS), terpenes, ribosomally synthesized and post-translationally modified peptides (RiPPs), arylpolyenes, phosphonates, and hydrogen cyanide-associated clusters. Notably, several clusters showed high similarity to known bioactive compounds. A siderophore-associated NRPS cluster displayed high similarity to pyochelin, while another NRPS-metallophore cluster corresponded to ornibactin variants (C4, C6, and C8), suggesting a strong capacity for iron acquisition. Additionally, a cluster with high similarity to pyrrolnitrin, a well-known antifungal compound, was identified, indicating potential antimicrobial activity. Other clusters included those related to terpene biosynthesis, and hybrid NRPS–PKS systems with similarity to myxochromide D. Several clusters exhibited low similarity to known compounds, including bolagladin-like and N-acyl amino acid-associated clusters, suggesting the presence of potentially novel secondary metabolites.

### 2.12. Protein–Protein Interactions Between Burkholderia and Rice

Using an interolog-based computational framework, we predicted putative protein–protein interaction networks between the three *Burkholderia* strains and *Oryza sativa japonica*. These predictions are based on sequence homology to experimentally validated interactions in model organisms.

#### 2.12.1. Putative PPIs Between *B. vietnamiensis* and Rice

**Figure 7:**
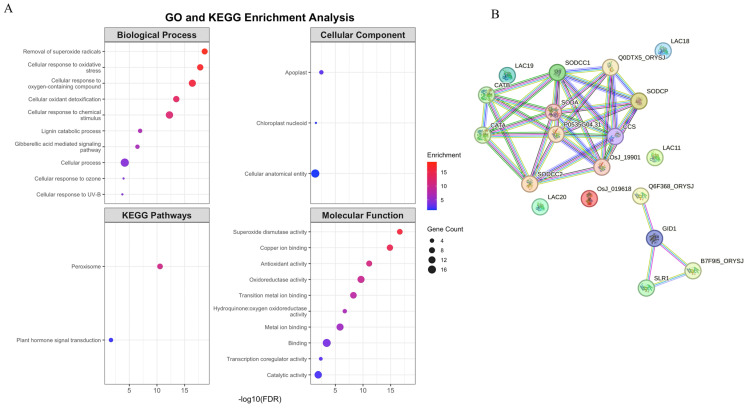
Functional enrichment analysis of *B. vietnamiensis*–*O. sativa japonica* protein–protein interactions. (**A**) GO and KEGG Enrichment analysis; Biological process enrichment highlighting oxidative stress-related functions and plant wall degradation; Molecular function enrichment, focusing on superoxide dismutase and antioxidant activities; Cellular component enrichment showing apoplast and chloroplast targeting; KEGG pathway enrichment, emphasizing peroxisomes and plant hormone signaling, (**B**) Protein–protein interaction network illustrating the connectivity of key proteins involved in oxidative stress and metabolic regulation.

Functional enrichment analysis of the *B. vietnamiensis*–*Oryza sativa japonica* putative PPI dataset identified multiple significantly enriched terms across Gene Ontology, KEGG (Figure 7), predominantly associated with oxidative stress response and reactive oxygen species (ROS) detoxification. This enrichment pattern indicates that oxidative stress management represents a central feature of the predicted interaction landscape.

The greatest enrichment, according to the biological processes identified in this dataset, was for the removal of superoxide radicals. Following this biological process were cellular responses to oxidative stress and cellular oxidant detoxification. In addition, the enrichment observed for the biological process of cellular response to oxygen-containing compounds also indicates a broader adaptation to oxidative stress. The lignin catabolic process indicates that these processes may also play a role in modifying the plant cell wall through the activity of laccase enzymes. There was also significant enrichment for environmental stress-related processes, such as the cellular response to ozone and ultraviolet-B radiation, indicating that these processes will assist in adaptation to these stresses.

The molecular function of superoxide dismutase was the most significantly enriched term, followed closely by copper ion binding and antioxidant activity. Oxidoreductase activity and hydroquinone:oxygen oxidoreductase activity (FDR = 1.96 × 10^−7^; 4 proteins) also point to oxidative enzymatic systems. Therefore, the analysis highlights the importance of copper-dependent enzymes, particularly superoxide dismutases and laccases, in mediating the detoxification and redox balance of reactive oxygen species.

Similarly, the analysis of cellular components demonstrates significant enrichment in the apoplast (FDR = 0.0034; 4 proteins), indicating that the vast majority of interactions occur within the extracellular space, especially at the plant/microbe interface. There was also extreme enrichment of components associated with chloroplasts, indicating that chloroplasts also participate as cellular compartments that generate reactive oxygen species (ROS). KEGG pathway analysis determined the peroxisome pathway to be the most significantly enriched pathway (FDR = 2.80 × 10^−11^; 7 proteins), supporting the peroxisome’s role in the metabolism of ROS; while the plant hormone signal transduction pathway (FDR = 0.0207; 4 proteins) was also significantly enriched and will assist in regulating growth/defense trade-offs as supported by the presence of gibberellin signaling components GID1 and SLR1. Protein–protein interaction clustering yielded a highly interconnected network in which superoxide dismutase-related proteins form the core functional unit, laccases related to lignin metabolism form a secondary cluster, and the copper chaperone proteins (CCS) are likely candidates for maintaining metal homeostasis throughout the network. Finally, peripheral nodes contain components involved in hormone signaling, resulting in possible regulatory integration within the interaction network. Based on these findings, we conclude that the interaction between *B. vietnamiensis* and rice is primarily mediated by oxidative stress mitigation, cell wall modification, and hormone regulation, resulting in a coordinated response to both environmental and biological stresses.

#### 2.12.2. Putative PPIs Between *B. gladioli* and Rice

**Figure 8:**
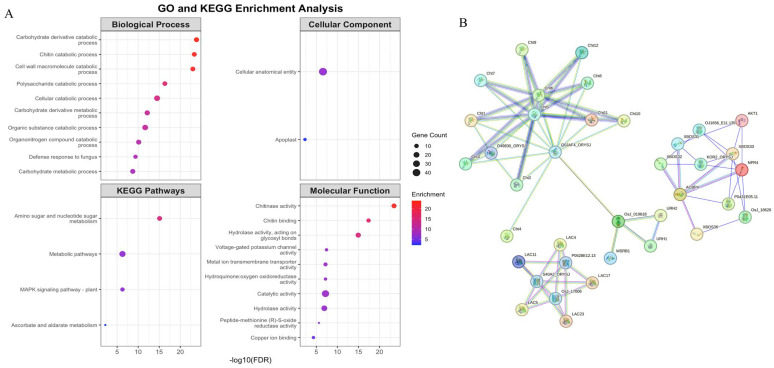
Functional enrichment and protein–protein interaction (PPI) network analysis of the *B. gladioli*–*O. sativa japonica* dataset. (**A**) Gene Ontology (GO) Biological Process enrichment showing significant overrepresentation of carbohydrate catabolic processes, including chitin and polysaccharide metabolism. GO Cellular Component enrichment indicating predominant localization in the apoplast, and cellular anatomical entity. GO Molecular Function enrichment highlighting chitinase activity and hydrolase functions acting on glycosyl bonds. KEGG pathway enrichment analysis demonstrating significant pathways such as amino sugar and nucleotide sugar metabolism, metabolic pathways, and plant signaling pathways. (**B**) Protein–protein interaction network illustrating functional clusters, including chitinase-related proteins, laccase-associated modules, signaling components, and metabolic regulators. Node size corresponds to the degree of connectivity, and edge thickness represents the interaction confidence.

Functional enrichment analysis of the *B. gladioli*–*O. sativa japonica* putative protein–protein interaction dataset revealed all the identified functional databases had multiple terms significantly enriched, predominantly related to carbohydrate metabolism and degradation of plant cell wall components (Figure 8), which indicates that these organisms potentially have a high level of functional specialization towards the use of polysaccharides and modification of host structural elements. Of note, when examining the biological processes, the most significantly enriched term was carbohydrate derivative catabolic process (FDR = 8.46 × 10^−25^; 15 protein), this was followed by chitin catabolic process (FDR = 2.29 × 10^−22^; 12 proteins) and cell wall macromolecule catabolic process (FDR = 5.02 × 10^−22^; 12 proteins). Enrichment was also observed for the polysaccharide catabolic process (FDR = 1.92 × 10^−15^; 12 proteins) and the cellular catabolic process (FDR = 8.03 × 10^−16^; 23 proteins), demonstrating that polysaccharides and complex structural polymers are subject to extensive degradation. The evidence presented strongly supports the involvement of carbohydrate active enzymes in the process of remodeling plant cell walls. When examining molecular function, primarily enriched terms were directly associated with hydrolase activity towards glycosidic bonds and chitinase activity, which is completely consistent with the observed breakdown of polysaccharides. The identification of oxidoreductase activity suggests that these carbohydrate-active enzymes may be involved in lignin modification via redox-based enzymatic mechanisms and may provide further adaptation in the metabolism of these organisms. When analyzing enrichment by cellular components, enrichment in the apoplast indicated that the majority of key reactions occur externally to the plant cell, in the region of the initial plant–microbe interaction. This supports the hypothesized requirement for secreted enzymes to degrade plant cell wall components. Protein interaction network analysis revealed clustering of carbohydrate-degrading enzymes into groups, including multiple chitinase-related proteins (i.e., members of the chitinase family forming a very tightly connected functional unit), which are part of the major carbohydrate-degrading enzyme cluster identified above. A secondary cluster of laccase-related proteins (e.g., LAC11 and LAC23) was also identified and was associated with lignin metabolism. Both clusters suggest that all carbohydrates being degraded and structurally modified are undergoing coordinated enzymatic activity.

Overall, these data indicate that the *B. gladioli*–*O. sativa japonica* interaction is largely mediated through carbohydrate metabolism and the enzymatic degradation of plant cell wall components; particularly, this interaction appears to be primarily alimentary in nature and reflects a strategy to facilitate nutrient acquisition from the host and structure interactivity with the host plant.

#### 2.12.3. Putative PPIs Between *B. cepacia* and Rice

**Figure 9:**
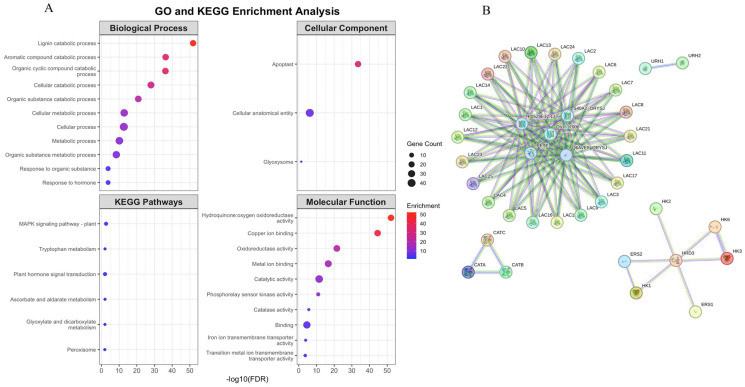
Functional enrichment analysis and protein–protein interaction (PPI) network of *B. cepacia* in *O. sativa japonica*. (**A**) shows the GO and KEGG enrichment analysis, Biological process (Gene Ontology) enrichment, highlights key metabolic and catabolic processes. Cellular component enrichment, focus on cellular localization. Molecular function enrichment, emphasize on oxidoreductase activities. KEGG pathway enrichment related to metabolic processes. (**B**) depicts the PPI network, showing interactions between key proteins involved in detoxification and metabolic pathways.

The analyses of the *B. cepacia*–*Oryza sativa japonica* putative protein–protein interaction dataset for functional enrichment revealed significant enrichments, largely within redox metabolism, detoxification processes, and the degradation of both aromatic and complex organic compounds (Figure 9). It was indicated by these results that a functional specialization toward oxidative metabolism and the adaptation of the plant-microbe interface toward oxidative stress. There was enrichment of biological processes in lignin catabolic processes as well as in aromatic compound degradation process pathways, indicating that enzymes were involved that could enable the modification of phenolic compounds derived from plants.

There was also enrichment of microbial catabolic processes (organic substances) and cellular catabolic processes, indicating a broad metabolic capacity to degrade substrates. The presence of catabolic processes for hydrogen peroxide provides additional evidence for a role(s) in the detoxification of reactive oxygen species (ROS). The oxidoreductase activity category is a major source of enrichment for molecular functions that correlate with specific redox reactions mediated by enzymes. The enrichment of hydroquinone:oxygen oxidoreductase activity and copper ion binding suggests that certain redox-active enzymes were present, including multicopper oxidases. Collectively, the laccase-like proteins (e.g., LAC11, LAC19, LAC20) identified within the interaction network are known to be involved in lignin modification and the oxidation of phenolic compounds. Collectively, they were observed to reinforce the predicted role(s) in the detoxification of hydrogen peroxide. In the cellular component analyses, there was enrichment for the apoplast, thereby indicating that key interactions occur at the extracellular plant-microbe interface. This supports the activities of secreted enzymes, such as laccases and oxidoreductases, which modify the host plant cell wall and detoxify plant-derived compounds.

In agreement with the above, pathway analyses from the KEGG database indicated enrichment of pathways linked to aromatic compound metabolism, glyoxylate and dicarboxylate metabolism, and peroxisome-associated processes. These three pathways are directly linked to oxidative metabolism and processes associated with oxidative stress response. Also supporting the above observations, protein–protein interaction network analyses identified numerous interconnected functional modules that support redox metabolism and stress adaptation processes. One of the principal clusters in this study consisted of oxidative stress-related enzymes, including the catalases (CATB and CATC), which are involved in the breakdown of hydrogen peroxide and cellular detoxification. A secondary supporting module included the laccase-like proteins (LAC11, LAC19, LAC20), which are multicopper oxidases involved in lignin modification and the oxidation of phenolic compounds. These observations support the enrichment of copper ion binding and oxidoreductase activity, suggesting a role(s) in the interactions associated with plant cell walls and extracellular redox-related processes. Another functional group included the following metabolic enzymes: hexokinase (HK3) and uridine ribohydrolase (URH1), which indicate an integration of primary metabolism and detoxification pathways. Collectively, these proteins have the potential to contribute to substrate use and metabolic flexibility during interactions with the host plant. Furthermore, some peripheral nodes identified in the above analyses were signaling-related components, including ethylene response sensors (ERS1 and ERS2), which are known to be associated with plant hormone signaling pathways. Collectively, these data indicate that there is likely a limited interface between the bacteria’s activity and the host plant’s regulatory systems.

## 3. Discussion

### 3.1. Genomic Streamlining in B. vietnamiensis for Beneficial Lifestyle

The non-pathogenic *B. vietnamiensis* J14EpLeaf2 possesses a substantially smaller genome (6.95 Mb) than the two pathogenic strains (8.36–8.46 Mb). This genome streamlining is consistent with observations in other beneficial plant-associated bacteria, where reductive evolution eliminates genes unnecessary for host-associated lifestyles [19,20]. The reduced number of genomic islands, prophages, and mobile genetic elements in *B. vietnamiensis* suggests fewer recent horizontal gene transfer events, potentially reflecting a stabilized mutualistic association with rice [21,22].

The absence of polysaccharide lyases (PLs) in *B. vietnamiensis* is particularly noteworthy. PLs are essential for pectin degradation, a key step in the maceration of plant cell walls by necrotrophic pathogens [23]. The lack of these enzymes in *B. vietnamiensis* is consistent with its non-pathogenic phenotype observed in plant bioassays [16] and suggests that this strain does not actively degrade intact plant cell walls—a critical safety feature for a biocontrol agent.

### 3.2. The Pathogenic Arsenal of B. gladioli and B. cepacia

*B. gladioli* harbor a complete T3SS apparatus and the most expanded T6SS gene cluster (14 genes) among the three strains. The T3SS is a hallmark of many Gram-negative pathogens, delivering effector proteins directly into host cells to suppress immune responses [24]. In contrast, *B. cepacia* lacks structural T3SS genes entirely, relying instead on T2SS and T6SS for protein secretion. Despite this, EffectiveT3 predicted 271 candidate effectors in *B. cepacia*—likely reflecting proteins secreted through alternative systems rather than a canonical T3SS. The substantially larger predicted effector repertoire in *B. gladioli* (301) compared to *B. vietnamiensis* (180) suggests a greater genomic capacity for host immune modulation, though functional studies are needed to confirm effector activity [25].

The expanded CAZyme repertoires in both pathogens—particularly GH and PL families—are consistent with an enhanced genomic potential to degrade plant cell walls for nutrient acquisition and tissue invasion. The presence of a complete Capsule I cluster in *B. gladioli* and a partial cluster in *B. cepacia* further supports their pathogenic potential, as capsules enhance survival in host environments and protect against phagocytosis [26,27].

The mobile genetic element profiles of the two pathogenic strains also differ in ways that reflect their distinct evolutionary trajectories. *B. gladioli* harbors the greatest number of prophages (5) among the three strains, which may account for a portion of its accessory genome diversity through phage-mediated horizontal gene transfer and lysogenic conversion. The high CRISPR loci count in *B. gladioli* (12, compared to 5 in *B. vietnamiensis* and 4 in *B. cepacia*) likely reflects a more active adaptive immune response to phage pressure, consistent with its elevated prophage burden and suggesting a history of frequent phage exposure. Together, these patterns indicate that *B. gladioli* occupies a dynamic genomic niche shaped by ongoing phage-host conflict, whereas *B. cepacia*’s genomic plasticity is driven more by genomic island acquisition (19 islands) than by prophage integration, and *B. vietnamiensis* shows consistently lower counts across both categories, consistent with the genome streamlining discussed in Section 3.1.

Importantly, however, the two pathogenic strains employ fundamentally distinct infection strategies that should not be conflated. *B. gladioli* represents a classical plant pathogen, deploying a complete T3SS, extensive phytotoxin gene repertoire (including *cysC1*, coronatine, and syringomycin-related genes), and a full Capsule I cluster—a molecular toolkit oriented toward host cell invasion, immune suppression, and tissue maceration [28,29]. *B. cepacia*, by contrast, lacks T3SS entirely and carries comparatively fewer phytotoxin-associated genes [30]. Its pathogenic capacity appears to derive instead from a broader resistome, expanded efflux systems, and—critically—the human-pathogen-associated markers cable pili and the ZmpA/ZmpB metalloproteases, which are absent in *B. gladioli* [17]. This distinction has practical implications: *B. gladioli* poses a primary risk as a plant pathogen, while *B. cepacia* poses a dual risk as both a plant pathogen and an opportunistic human pathogen of particular concern in immunocompromised populations [31,32]. Treating these strains as equivalent threats based on shared genome size or CAZyme counts would obscure this clinically relevant difference.

### 3.3. Biosafety Implications: The Case of B. cepacia J14Eple

The presence of cable pili and ZmpA/ZmpB metalloprotease genes in *B. cepacia* J14Eple is the most significant biosafety finding from this study. Cable pili are characteristic of epidemic strains of the *B. cepacia* complex that infect cystic fibrosis (CF) patients, with associated increases in person-to-person spread and rapid declines in the clinical status of CF individuals (the so-called “cepacia syndrome”) [33]. In addition, ZmpA and ZmpB are zinc metalloproteases that degrade host proteins, thereby enhancing pathogenicity [34].

The contrasting plant bioassay results reported in the companion study [16] are consistent with the genomic profiles established here. The absence of visible disease symptoms in rice and tobacco inoculated with *B. vietnamiensis* J14EpLeaf2 is consistent with its lack of a complete T3SS, absence of the Capsule I cluster, and minimal phytotoxin-associated gene content—the principal determinants of plant cell invasion and immune suppression that are present in both pathogenic strains. This genomic-phenotypic concordance strengthens the reliability of genome-based biosafety assessments for candidate biocontrol strains and underscores the value of comparative genomics as a pre-screening tool prior to plant inoculation trials.

Despite its antifungal activity, this strain carries genetic markers of high-risk opportunistic pathogenicity. Regulatory approval for field applications would be unlikely, and its use would pose unacceptable risks to immunocompromised individuals [35]. In contrast, *B. vietnamiensis* J14EpLeaf2 lacks these markers. While it possesses intrinsic efflux pumps and beta-lactamases (typical of environmental *Burkholderia*), it does not carry the epidemic-associated virulence determinants found in *B. cepacia*. This supports its continued development as a biocontrol agent, though strain-level biosafety testing (including animal infection models) should be completed before field release [5].

### 3.4. Secondary Metabolites, Biocontrol and Plant Growth Promotion

The use of LC-MS metabolomics yielded successful verification for the presence of a number of antiSMASH predicted BGCs (Biosynthetic Gene Clusters) present in *B. vietnamiensis* and *B. gladioli*. The identification of IAA (Indole-3-Acetic Acid) in both species provides evidence of their auxiliary biosynthetic capacity, which could help promote plant growth. The presence of IAA in greater quantities in *B. vietnamiensis* has been shown to correlate with its beneficial plant phenotype [16].

The identification of numerous metabolites connected to ornibactin and those associated with AHLs indicates that *B. vietnamiensis* is capable of active secondary metabolism. The finding of a penicillin V acylase-like protein exclusively in *B. vietnamiensis* provides the opportunity to explore a novel form of quorum quenching. A functionally confirmed penicillin V acylase from *B. vietnamiensis* could degrade AHL signals made by competing fungi, or bacteria, contributing to biocontrol, as similarly demonstrated in other bacteria that live on plants [36,37]. The presence of enacyloxins, such as virginiamycin M1, and occidiofungins, such as antibiotic M83, provides a mechanism for the aforementioned antifungal activity demonstrated by *B. vietnamiensis* against R. solani (77% inhibition [16]). These compounds warrant further investigation as leads for biopesticide development.

A notable finding is that *B. gladioli*, despite being a plant pathogen, also produces antifungal compounds validated by LC-MS, including virginiamycin M1 and multiple siderophores. This is consistent with the broader observation that pathogenic and biocontrol functions are not mutually exclusive at the genomic level—the same iron acquisition and antimicrobial machinery that confers competitive fitness in the phyllosphere may also contribute to virulence depending on host context and environmental conditions [38]. This underscores why phenotypic antifungal screening alone is insufficient for strain selection in biocontrol development; comprehensive genomic and biosafety characterization are essential complements to activity-based assays.

Genome mining of *B. cepacia* using antiSMASH identified diverse predicted BGCs spanning NRPS, PKS, terpene, RiPP, arylpolyene, phosphonate, and hydrogen cyanide-associated classes. It is important to note that these remain genomic predictions—*B. cepacia* was excluded from LC-MS metabolite profiling in this study, given its early designation as a biosafety risk, which precluded its consideration as a biocontrol candidate. Consequently, whether these BGCs are expressed and whether the predicted compounds are produced under relevant conditions remains unknown. The presence of siderophore-producing clusters such as pyochelin and ornibactin reflects a strong genomic capacity for iron acquisition, which is critical for survival in competitive environments such as the plant phyllosphere [39]; similar siderophore systems have been widely reported in *Burkholderia* species and are often associated with both environmental fitness and virulence [40]. The predicted pyrrolnitrin biosynthetic genes suggest potential antifungal activity that may contribute to microbial competition [41], though whether such compounds are produced and at what concentrations remains to be determined. The presence of hydrogen cyanide-associated clusters, would raise additional biosafety considerations given the cytotoxic potential of HCN at elevated concentrations [42]. Several clusters showed low similarity to known compounds, suggesting the presence of novel biosynthetic pathways warranting further characterization [43].

Collectively, the predicted BGC repertoire of *B. cepacia* reflects metabolic versatility consistent with its ecological adaptability but does not alter the biosafety assessment based on its virulence factor profile. This dual functionality reinforces the importance of careful strain-level evaluation before any consideration of agricultural application.

### 3.5. Putative Protein–Protein Interaction Network Suggest Distinct Interaction Strategies

The computational PPI network analysis was conducted to provide a systems-level perspective that complements the individual genomic analyses presented above. All predicted interactions are computationally inferred using the interolog approach, based on sequence homology to experimentally validated interactions in model organisms, and require experimental validation before definitive biological conclusions can be drawn. The principal value of this analysis lies not in generating independent findings, but in integrating the secretion system, CAZyme, virulence factor, and secondary metabolite data into a network-level framework that reveals the functional connectivity and predicted interaction architecture of each strain.

*B. gladioli* and *B. cepacia* displayed denser predicted interaction networks with a higher number of hub proteins compared to *B. vietnamiensis*, consistent with the broader genomic evidence for more complex host interaction repertoires in pathogenic strains. The enrichment of T6SS-associated hub proteins in *B. gladioli* aligns with its expanded T6SS gene repertoire (14 genes) and is consistent with a role for this system in both interbacterial competition and host cell targeting [44], functions that are mechanistically linked in many Gram-negative pathogens where T6SS serves as a dual-purpose weapon against competing bacteria and eukaryotic host cells. The predicted clustering of chitinase and carbohydrate catabolism hubs in *B. gladioli* further reinforces the CAZyme data, suggesting that cell wall-degrading enzymes do not operate in isolation but form coordinated functional modules at the host–pathogen interface [45,46]. When the analysis was restricted to predicted secreted proteins and effector candidates, pathogenic strains showed a significantly higher proportion of hub nodes than *B. vietnamiensis*, consistent with their expanded predicted effector repertoires (301 in *B. gladioli* and 271 in *B. cepacia* vs. 180 in *B. vietnamiensis*). For *B. cepacia*, these hub candidates likely represent proteins exported through T2SS or T6SS rather than a canonical T3SS, given the complete absence of structural T3SS genes in this strain [47].

In *B. vietnamiensis*, the predicted network was more modular and less densely connected, with hubs enriched in nutrient transport (ABC transporters), siderophore biosynthesis, and quorum sensing—a topology consistent with a cooperative lifestyle oriented toward metabolic exchange and population-level coordination rather than host manipulation [48,49]. The predicted enrichment of superoxide dismutase (SOD) activity at the *B. vietnamiensis*–rice interface is particularly noteworthy [50]. ROS detoxification is a well-documented hallmark of beneficial endophytes, enabling persistent plant colonization by neutralizing the oxidative burst that plants deploy as a frontline immune response [51]. The network-level prediction of SOD enrichment therefore not only aligns with the genomic and metabolomic evidence for *B. vietnamiensis* as a non-invasive strain, but provides a plausible molecular mechanism by which it may suppress host immune activation during colonization [51]—a hypothesis that warrants direct experimental testing through ROS assay and plant immune marker profiling.

In *B. cepacia*, the predicted PPI network revealed catalase isoforms (CATB, CATC) forming a central oxidative stress module [52], alongside metabolic enzymes including hexokinases (HK1–HK6) and uridine ribohydrolases (URH1/URH2), indicating predicted integration of redox detoxification with primary carbon metabolism and nucleotide turnover. This metabolic architecture suggests a strain capable of sustained activity under oxidative stress conditions—consistent with its expanded resistome and the broad environmental adaptability inferred from its BGC repertoire. The presence of ethylene response sensor components (ERS1/ERS2) as peripheral nodes is intriguing: ethylene signaling governs both plant defense activation and senescence, and its predicted engagement by *B. cepacia*-associated proteins raises the hypothesis that this strain may modulate host hormonal balance during infection, potentially suppressing defense responses or accelerating tissue senescence to facilitate colonization [53]. This remains speculative at the network prediction level but represents a tractable hypothesis for future experimental investigation using ethylene signaling mutants.

Taken together, the findings of this study demonstrate that lifestyle divergence among rice-associated *Burkholderia* strains cannot be reduced to a simple pathogen-versus-beneficial binary. *B. gladioli* and *B. cepacia* share pathogenic outcomes but achieve them through divergent molecular strategies—the former through classical plant-pathogen machinery including T3SS, phytotoxins, and capsule biosynthesis, the latter through alternative secretion systems, an expanded resistome, and epidemic-associated human virulence determinants. Meanwhile, *B. gladioli* produce validated antifungal compounds despite its pathogenic designation, illustrating that biocontrol-relevant metabolic capacity and virulence determinants can co-exist within a single genome. These observations collectively argue that strain-level genomic characterization—rather than species-level classification or activity-based screening alone—is the appropriate framework for evaluating *Burkholderia* strains for agricultural applications. The discovery of cable pili and ZmpA/ZmpB in a rice-isolated *B. cepacia* strain further suggests that environmental *Burkholderia* screening programs should incorporate routine surveillance for epidemic-associated markers of human pathogenicity, regardless of antifungal phenotype. Several limitations of this study should be acknowledged: genome assemblies remain at scaffold level, analyses are based on single representative strains per species, PPI predictions are computationally inferred and require experimental validation, and the biocontrol efficacy of *B. vietnamiensis* under field conditions remains to be demonstrated. Future work should prioritize complete genome closure, in planta validation of key predicted effectors and secondary metabolites, and progression of *B. vietnamiensis* J14EpLeaf2 through structured biosafety and field efficacy trials.

## 4. Materials and Methods

### 4.1. Bacterial Strains

Three *Burkholderia* species isolated from the phyllosphere of rice plants were used: *Burkholderia cepacia* strain J14Eple, *Burkholderia vietnamiensis* strain J14EpLeaf2, and *Burkholderia gladioli* strain A1EpSeed5. All strains demonstrated inhibitory effects against *Rhizoctonia solani* AG1-IA but exhibited different interactions with rice plants, as detailed in the companion study [16].

### 4.2. DNA Extraction, Library Construction and Sequencing

Genomic DNA from three bacterial strains was extracted using a bacterial DNA extraction kit (Sangon Biotech, Shanghai, China) following the manufacturer’s instructions. For each sample, 1 µg of high-quality genomic DNA was used as input for library construction. Sequencing libraries were prepared using the NEBNext Ultra DNA Library Prep Kit for Illumina (New England Biolabs, Ipswich, MA, USA), and index adapters were added to enable sample multiplexing. Briefly, genomic DNA was fragmented by sonication to an average size of approximately 350 bp, followed by end repair, A-tailing, and ligation of full-length Illumina adapters, with subsequent PCR amplification. PCR products were purified using the AMPure XP system (Beckman Coulter, Brea, CA, USA), and library size distribution was assessed using an Agilent 2100 Bioanalyzer (Agilent Technologies, Santa Clara, CA, USA). Libraries were quantified by real-time PCR prior to sequencing. Whole-genome sequencing was performed on an Illumina NovaSeq PE150 platform at Beijing Novogene Bioinformatics Technology Co., Ltd. (Beijing, China).

### 4.3. Data Processing and Assembly

Clean reads were obtained using readfq (version 10) to remove low-quality sequences. Reads were discarded if they contained more than 40% low-quality bases (quality score ≤ 20), more than 10% ambiguous bases (N), or adapter contamination exceeding 15 bp. Prior to genome assembly, genome size was estimated using a k-mer-based approach. Clean reads were independently assembled using SOAPdenovo (version 2.04), SPAdes, and ABySS [54,55,56]. The resulting assemblies were integrated using CISA, and the assembly with the fewest scaffolds was selected as the optimal assembly. GapCloser (version 1.12) was then applied to fill gaps. Contigs shorter than 500 bp were removed, and the final high-quality assembly was used for subsequent analyses.

### 4.4. Genome Component and Function Prediction

Protein-coding genes were identified using GeneMarkS (version 4.17) [57]. Transfer RNA (tRNA) genes were predicted with tRNAscan-SE (version 1.3.1) [58], and ribosomal RNA (rRNA) genes were identified using RNAmmer (version 1.2) [59]. Genomic islands were predicted using IslandPath-DIMOB (version 0.2) [60]. Prophage regions were identified using phiSpy (version 2.3) [61], and CRISPR arrays were predicted using CRISPRdigger (version 1.0) [62]. Functional annotation of predicted genes was performed by searching against GO, KEGG [63], COG [64], NR [65], TCDB [66], and Swiss-Prot databases (E-value threshold 1 × 10^−5^, minimum coverage 40%).

### 4.5. Secretion Systems and Effector Prediction

Signal peptides were predicted using SignalP v4.0 [67], and transmembrane domains using TMHMM v2.0c. Proteins associated with secretion systems were identified using EffectiveT3 [68]. Type III secretion system (T3SS) effector proteins were further predicted using Effective T3 v1.0.1 [69].

### 4.6. Secondary Metabolite Gene Clusters

Secondary metabolite biosynthetic gene clusters were identified using antiSMASH (version 8.0.2) [70].

### 4.7. Virulence Factor and Pathogenicity Analysis

Virulence factor genes were identified by searching against the Virulence Factors of Pathogenic Bacteria Database (VFDB) [71]. Pathogen-host interaction genes were annotated using PHI-base [72]. Antibiotic resistance genes were identified using CARD [73].

### 4.8. Carbohydrate-Active Enzyme Analysis

Carbohydrate-active enzymes (CAZymes) were annotated using the CAZy database [74], including glycoside hydrolases (GH), glycosyltransferases (GT), polysaccharide lyases (PL), carbohydrate esterases (CE), auxiliary activities (AA), and carbohydrate-binding modules (CBM).

### 4.9. LC-MS Metabolite Profiling

Sample preparation, UPLC separation, and mass spectrometry were performed as described in the companion study [16]. Metabolites were identified by comparison against HMDB, KEGG, and PubChem databases.

### 4.10. Protein–Protein Interaction (PPI) Analysis

Protein–protein interaction (PPI) networks between *Burkholderia* strains and rice (*Oryza sativa*) were predicted using an interolog-based computational framework. This approach infers protein–protein interactions from sequence homology to experimentally validated or predicted interactions in model organisms.

Protein sequences from the three *Burkholderia* genomes (*B. vietnamiensis* J14EpLeaf2, *B. gladioli* A1EpSeed5, and *B. cepacia* J14Eple) were obtained from the annotated proteomes generated in this study. The reference proteome of *Oryza sativa japonica* was retrieved from UniProtKB and Phytozome databases.

To predict cross-species PPIs between *Burkholderia* and rice, we used an interolog approach: known protein–protein interactions from model organisms (retrieved from STRING v12.0) were transferred to homologous protein pairs between each *Burkholderia* strain and *O. sativa japonica*. Homology was established using BLAST+ (version 2.14.0) with criteria of E-value ≤ 1 × 10^−5^, sequence identity ≥30%, and coverage ≥40%. Only reciprocal best hits were retained as orthologs.

Predicted interaction datasets were further analyzed using STRING (version 12.0), integrating both known and predicted functional associations. Interaction networks were constructed with a medium-to-high confidence threshold (score ≥ 0.7) to ensure reliability. First-shell interactors (direct interaction partners of seed proteins) were retained to limit network complexity while preserving biologically meaningful connections.

Network visualization and topological analysis were performed using Cytoscape (version 3.10). Node size was scaled according to degree centrality (number of connections per node), and hub proteins were defined as nodes with ≥15 interactions. Edge thickness corresponded to interaction confidence scores (higher confidence = thicker edges).

Functional enrichment analysis of network proteins was conducted using g:Profiler (version 0.2.3) (gost, FDR < 0.05, gSCS correction for multiple testing). Gene Ontology (GO) terms (Biological Process, Molecular Function, and Cellular Component) and KEGG pathways were tested for overrepresentation. KEGG pathway mapping was performed using KAAS (version 2.1) (KEGG Automatic Annotation Server, cutoff 60%), followed by enrichment analysis with KOBAS 3.0 (FDR < 0.05, hypergeometric test).

To enhance biological significance, analyses concentrated mainly on predicted secreted proteins and effector candidates identified through SignalP v4.1 (signal peptides), TMHMM v2.0c (transmembrane domains), and secretion system annotation (EffectiveT3), as these proteins are more likely to participate in host–microbe interactions.

### 4.11. Nucleotide Sequence Accession Numbers

The whole-genome sequences have been deposited in NCBI under accession numbers SAMN24971777 (*B. cepacia* J14Eple), SAMN24971778 (*B. vietnamiensis* J14EpLeaf2), and SAMN24971779 (*B. gladioli* A1EpSeed5).

## 5. Conclusions

This study provides a comprehensive genomic framework for distinguishing pathogenic from beneficial *Burkholderia* strains isolated from the same ecological niche. Several key discriminative features were identified. Pathogenic strains (*B. gladioli* and *B. cepacia*) possess larger genomes (8.36–8.46 Mb) enriched in mobile genetic elements and expanded secretion system repertoires. Notably, *B. gladioli* harbors a complete T3SS apparatus, while *B. cepacia* relies on T2SS and T6SS in the absence of structural T3SS genes. Both pathogenic strains possess expanded CAZyme repertoires (including polysaccharide lyases), Capsule I-associated genes, and phytotoxin-related determinants. In contrast, the beneficial strain *B. vietnamiensis* J14EpLeaf2 exhibits a streamlined genome (6.95 Mb), lacks polysaccharide lyases and a complete T3SS, and does not harbor major virulence-associated markers.

From a biosafety perspective, *B. cepacia* J14Eple carries epidemic-associated determinants, including cable pili and ZmpA/ZmpB metalloproteases, which are linked to opportunistic pathogenicity. These features raise significant safety concerns and may limit its suitability for agricultural application, underscoring the importance of strain-level risk assessment. In contrast, *B. vietnamiensis* J14EpLeaf2 lacks these high-risk markers and produces plant-beneficial metabolites, including indole-3-acetic acid (IAA) and antifungal compounds such as occidiofungin A and cepacin A. The presence of a putative penicillin V acylase homolog further suggests a potential quorum-quenching capability, although this requires experimental validation.

Comparative PPI network analysis revealed that pathogenic strains tend to exhibit more complex and densely connected interaction networks, consistent with broader host interaction potential. In contrast, *B. vietnamiensis* displayed a more modular and streamlined network architecture, enriched in functions associated with oxidative stress mitigation, nutrient exchange, and metabolic coordination, supporting a cooperative plant-associated lifestyle.

This study demonstrates that integrating comparative genomics, metabolomics, and computational interaction modeling provides an effective strategy for differentiating beneficial strains from opportunistic pathogens. *B. vietnamiensis* J14EpLeaf2 emerges as a promising biocontrol agent against rice sheath blight caused by *Rhizoctonia solani*, as demonstrated in our companion study, which provides experimental validation of its efficacy and biosafety.

## Figures and Tables

**Table 1:** Genome assembly statistics for three *Burkholderia* species.

Species	Assembly Level	Total Number (>500 bp)	Total Length (bp)	N50 Length (bp)	N90 Length (bp)	Max Length (bp)	Min Length (bp)	GC (%)
*B. gladioli* A1EpSeed5	Scaffold	140	8,359,450	141,738	39,552	558,349	611	67.9
	Contig	183	8,359,020	106,813	28,144	558,349	611	67.9
*B. vietnamiensis* J14EpLeaf2	Scaffold	94	6,945,072	163,265	59,715	440,106	531	66.98
	Contig	110	6,944,912	120,032	46,992	361,124	531	66.98
*B. cepacia* J14Eple	Scaffold	56	8,458,640	335,300	108,060	604,613	579	66.84
	Contig	75	8,458,450	229,239	72,032	544,164	579	66.84

**Table 2:** Mobile Genetic Elements in *Burkholderia* strains.

Species	Genomic Islands	Prophages	CRISPR Loci
*B. gladioli*	14	5	12
*B. vietnamiensis*	12	2	5
*B. cepacia*	19	2	4

Full details of ncRNAs and additional statistics are provided in Supplementary Table S1.

**Table 3:** Secretion system gene counts in rice-associated *Burkholderia* strains.

Species	T1SS	T2SS	T3SS	T4SS	T6SS	Predicted T3SS Effectors
*B. gladioli*	1	7	5	2	14	301
*B. vietnamiensis*	0	12	7	14	8	180
*B. cepacia*	0	5	0	2	6	271

Gene counts represent the number of genes assigned to each secretion system type. Predicted T3SS effectors were identified using EffectiveT3 v1.0.1 based on N-terminal sequence features; for *B. cepacia*, which lacks structural T3SS genes, these candidates are likely secreted through alternative pathways. T1SS: Type I secretion system; T2SS: Type II; T3SS: Type III; T4SS: Type IV; T6SS: Type VI.

**Table 4:** Quorum Sensing and Quenching Genes.

Enzymes	*B. vietnamiensis*	*B. gladioli*	*B. cepacia*
Penicillin V acylase	+	−	−
AHL synthases	+	+	+
LuxR regulators	+	+	+

Symbols denote relative representation: +, present; −, not detected.

**Table 5:** Key Virulence factors in *Burkholderia* species.

Virulence Factor Category	*B. vietnamiensis*	*B. gladioli*	*B. cepacia*
Capsule I cluster (complete)	−	+ (20+ genes)	+/partial
LOS biosynthesis	+	+ (complete)	+ (complete)
T3SS (complete apparatus)	−	+	−
T6SS	+	++ (multiple clusters)	++ (expanded)
Phytotoxin genes (e.g., *cysC1*)	minimal	extensive	moderate−extensive
Urease	−	+	+/variable
Metalloproteases (e.g., ZmpB)	−	+	+
Type IV pili	+	+	+
Flagella/chemotaxis	+++	+++	+++
Iron uptake systems	+++	+++	+++

Symbols denote relative representation: +++, high; ++, moderate; +, present; +/partial, incomplete; −, not detected.

**Table 6:** Opportunistic pathogenicity Markers.

Marker	*B. vietnamiensis*	*B. gladioli*	*B. cepacia*
Cable pili	−	−	+
ZmpA/ZmpB metalloproteases	−	−	+
VgrG-5	−	−	−
SodC	−	−	−
CF-associated O-antigen	+	+	+

Symbols denote relative representation: +, present; −, not detected.

**Table 7:** Key Metabolites Identified in *Burkholderia vietnamiensis*.

(**A**) **Plant Growth Promotion and Auxin Biosynthesis**					
**Metabolite ID**	**Metabolite Name**	***m*/*z* Value**	**RT**	**Metabolite Class**	**Biological Role/Activity**
3.66_203.0831*m*/*z*	L-Tryptophan (ID 6305)	203.0831	3.66 min	Amino acid	IAA precursor—Essential starting material for auxin biosynthesis
5.24_174.0564*m*/*z*	Indoleacetic Acid (IAA) (ID 802)	174.0564	5.24 min	Indole derivative	Primary plant growth hormone—Stimulates root development, cell division
4.92_205.0735*m*/*z*	Indolelactic Acid (ID 92904)	205.0735	4.92 min	Indole derivative	IAA pathway intermediate—Confirms active auxin biosynthesis
4.39_336.1549*m*/*z*	Pro Tyr Gly	336.1549	4.39 min	Peptide	Plant growth promotion
0.88_432.2206*m*/*z*	Shinflavanone	432.2206	0.88 min	Flavonoid	Plant-associated signaling
(**B**) **Quorum-Sensing Signal**					
**Metabolite ID**	**Metabolite Name**	***m*/*z* Value**	**RT**	**Metabolite Class**	**Biological Role/Activity**
6.09_272.1869*m*/*z*	C10-Hsl (ID 10131281)	272.1869	6.09 min	Organic acid derivative	Quorum sensing signal—Population-dependent gene regulation
6.81_314.2343*m*/*z*	C12-Hsl (ID 10221437)	314.2343	6.81 min	Organic acid derivative	Quorum sensing signal—Coordinates group behaviors
(**C**) **Antimicrobial and Biocontrol Compounds**					
**Metabolite ID**	**Metabolite Name**	***m*/*z* Value**	**RT**	**Metabolite Class**	**Biological Role/Activity**
1.15_84.0215n	Penitricin D	175.0252	1.15 min	Antibiotic	Antimicrobial
4.56_466.1702*m*/*z*	Antibiotic M83	466.1702	4.56 min	Antibiotic	Antimicrobial
5.25_375.2487*m*/*z*	16-Kaurene-2,3,15-Triol	375.2487	5.25 min	Terpenoid	Antimicrobial
5.12_434.1949*m*/*z*	Fluvi-Moins-Dipox	434.1949	5.12 min	Lipid	Antimicrobial
4.96_284.1391*m*/*z*	Brevianamide F	284.1391	4.96 min	Diketopiperazine	Bioactive peptide
(**D**) **Siderophore**					
**Metabolite ID**	**Metabolite Name**	***m*/*z* Value**	**RT**	**Metabolite Class**	**Biological Role/Activity**
0.93_174.1003n	N(5)-Acetyl-L-Ornithine	175.1077	0.93 min	Amino acid derivative	Hydroxamate siderophore precursor
0.72_148.0842n	L-N5-Oh-Ornithine	212.1005	0.72 min	Amino acid derivative	Hydroxamate siderophore precursor
4.90_151.0403*m*/*z*	Vanillin	151.0403	4.90 min	Phenol	Catechol siderophore precursor
4.65_181.0510*m*/*z*	Hydroxyphenyllactic Acid	181.0510	4.65 min	Organic acid	Phenyllactate scaffold
(**E**) **Other Bioactive**					
**Metabolite ID**	**Metabolite Name**	***m*/*z* Value**	**RT**	**Metabolite Class**	**Biological Role/Activity**
5.92_318.2996*m*/*z*	Phytosphingosine	318.2996	5.92 min	Sphingolipid	Cell signaling
5.81_131.0715*m*/*z*	5-Oh-Caproic Acid	131.0715	5.81 min	Organic acid	Antifungal
4.52_230.1036*m*/*z*	Kainic Acid	230.1036	4.52 min	Organic acid	Neuroactive, toxic
1.26_263.0706*m*/*z*	Cysteinyl-Proline	263.0706	1.26 min	Peptide	Antioxidant

N means neutral mass.

**Table 8:** Key Metabolites Identified in *B. gladioli*.

(**A**) **Siderophores**					
**Metabolite ID**	**Metabolite Name**	***m*/*z* Value**	**RT**	**Metabolite Class**	**Biological Role/Activity**
1.04_448.1673*m*/*z*	Staphyloferrin B (ID 134820292)	448.1673	1.04 min	Peptide conjugate	Siderophore—Iron acquisition, virulence factor
1.66_451.1483*m*/*z*	Acinetobactin (ID 139588300)	451.1483	1.66 min	Peptide-phenol	Siderophore—Catechol-type iron chelator
1.22_529.2274*m*/*z*	Vulnibactin **3** (ID 139588390)	529.2274	1.22 min	Peptide-phenol	Siderophore—Iron acquisition, competitive fitness
1.39_485.2016*m*/*z*	Glucosylgalactosylhydroxylysine (ID 122304)	485.2016	1.39 min	Glycopeptide	Siderophore-related—Glycosylated iron binder
(**B**) **Antimicrobial/Antibiotic**					
**Metabolite ID**	**Metabolite Name**	***m*/*z* Value**	**RT**	**Metabolite Class**	**Biological Role/Activity**
4.07_525.2478n	Virginiamycin M1 (ID 5459319)	544.2662	4.07 min	Macrolide	Antibiotic—Streptogramin class, protein synthesis inhibitor
5.65_269.0458*m*/*z*	Genistein (ID 5280961)	269.0458	5.65 min	Isoflavonoid	Antimicrobial, phytoalexin, eukaryotic signaling
4.03_539.2941*m*/*z*	Cucurbitacin D (ID 5281318)	539.2941	4.03 min	Triterpenoid	Antimicrobial, cytotoxic, plant defense compound
5.09_404.1927*m*/*z*	Latrunculin A (ID 445420)	404.1927	5.09 min	Macrolide	Antimicrobial, actin polymerization inhibitor
4.28_526.2807*m*/*z*	Cytochalasin D (ID 6433799)	526.2807	4.28 min	Alkaloid	Antimicrobial, cytoskeleton disruptor
5.29_253.0510*m*/*z*	**3ebo** (ID 5281607)	253.0506	5.29 min	Flavonoid	Antimicrobial, antioxidant
4.94_441.1485*m*/*z*	Xanthohumol E (ID 25245324)	441.1485	4.94 min	Chalcone	Antimicrobial, anticancer, anti-inflammatory
(**C**) **Plant Growth Promotion**					
**Metabolite ID**	**Metabolite Name**	***m*/*z* Value**	**RT**	**Metabolite Class**	**Biological Role/Activity**
1.12_180.0669*m*/*z*	Tyrosine (ID 6057)	180.0669	1.12 min	Amino acid	Protein synthesis, IAA precursor (minor pathway)
0.80_146.0460*m*/*z*	Glutamic Acid (ID 33032)	146.0460	0.80 min	Amino acid	Primary metabolism, signaling
5.38_180.1513n	(3z,6z,9z)-Dodecatrienol (ID 5281129)	198.1851	5.38 min	Alcohol	Plant volatile, signaling
5.50_173.1187*m*/*z*	Hydroxynonanoic Acid (ID 5282897)	173.1187	5.50 min	Organic acid	Plant defense signaling
(**D**) **IAA (Auxin Pathway)**					
**Metabolite ID**	**Metabolite Name**	***m*/*z* Value**	**RT**	**Metabolite Class**	**Biological Role/Activity**
3.66_203.0831*m*/*z*	**L-Tryptophan** (ID 6305)	203.0831	3.66 min	Amino acid	IAA precursor
5.24_174.0564*m*/*z*	**Indoleacetic Acid (IAA)** (ID 802)	174.0564	5.24 min	Indole derivative	**Plant growth hormone**—Present but lower abundance than *B. vietnamiensis*
4.91_175.0633n	**2-(1h-Indol-3-Yl)Acetate** (ID 801)	176.0706	4.91 min	Indole derivative	IAA (ionized form)
4.22_203.0830*m*/*z*	**3h-Tryptophan** (ID 12998164)	203.0830	4.22 min	Indole derivative	Tryptophan-related
(**E**) **Other Bioactive Metabolite**					
**Metabolite ID**	**Metabolite Name**	***m*/*z* Value**	**RT**	**Metabolite Class**	**Biological Role/Activity**
5.92_318.2996*m*/*z*	**Phytosphingosine** (ID 122121)	318.2996	5.92 min	Sphingolipid	Cell signaling, membrane structure
4.96_284.1391*m*/*z*	**Brevianamide F** (ID 181567)	284.1391	4.96 min	Diketopiperazine	Bioactive peptide, antimicrobial
1.66_113.0470n	**Cyclo(Glycylprolyl)** (ID 193540)	155.0815	1.66 min	Diketopiperazine	Bioactive peptide, quorum sensing mimic

N means neutral mass.

## Data Availability

The original contributions presented in this study are included in the article and Supplementary Materials. Further inquiries can be directed to the corresponding author.

## Supplementary Materials

The following supporting information can be downloaded at: https://www.mdpi.com/article/10.3390/ijms27114730/s1.
